# Comparative Transcriptome Profiling of Cold Exposure and β3-AR Agonist CL316,243-Induced Browning of White Fat

**DOI:** 10.3389/fphys.2021.667698

**Published:** 2021-05-04

**Authors:** Yu Li, Xiaodan Ping, Yankang Zhang, Guoqiang Li, Ting Zhang, Geng Chen, Xinran Ma, Dongmei Wang, Lingyan Xu

**Affiliations:** Shanghai Key Laboratory of Regulatory Biology, School of Life Sciences, Institute of Biomedical Sciences, East China Normal University, Shanghai, China

**Keywords:** browning of white fat, cold exposure, β3-AR agonist, heterogeneity analysis, transcriptomics

## Abstract

Beige adipocytes are newly identified thermogenic-poised adipocytes that could be activated by cold or β3-adrenergic receptor (β3-AR) signaling and offer therapeutic potential for treating obesity and metabolic diseases. Here we applied RNA-sequencing analysis in the beige fat of mice under cold exposure or β3-AR agonist CL316,243 (CL) treatment to provide a comparative and comprehensive analysis for the similarity and heterogeneity of these two stimulants. Importantly, via KEGG analysis, we found that cold and CL commonly induced oxidative phosphorylation. Meanwhile, cold increased glycerolipid and amino acids metabolism while CL treatment triggered a broader spectrum of metabolic responses including carbohydrate metabolism. Besides, cold or CL treatment featured greater heterogeneity in downregulated gene programs. Of note, the top changed genes in each category were confirmed by qPCR analysis. Overall, our analysis provided a better understanding of the heterogeneity of differential models for beige adipocytes activation and a possible clue for optimizing β3-AR agonists in the future.

## Introduction

Obesity, manifested as excess fat accumulation caused by the imbalance between energy intake and expenditure, is a severe public health crisis throughout the world since it is the major risk factor for metabolic diseases including type 2 diabetes, hypertension, cardiovascular disease, and certain types of cancers (Harms and Seale, 2013). Fat tissues are divided into three categories. White fat stores energy in the form of triglyceride and classic brown fat dissipates chemical energy as heat via uncoupling protein 1 (UCP1). Recently, beige adipocytes have been discovered and characterized by their high thermogenic and energy dissipating capacity upon cold exposure or β3-adrenergic signaling (Himms-Hagen et al., 1994; Rosell et al., 2014), which is called the “browning of white fat.” Of note, the browning phenomenon was also observed in cold-exposed human adults in the supraclavicular region revealed by PET-CT scans with characteristics resembling beige/brown adipocytes in rodents (Nedergaard et al., 2007; Sharp et al., 2012). The existence of beige/brown fat in human adults has attracted great attention and has been considered a novel peripheral target to treat obesity and metabolic diseases.

Under cold exposure, norepinephrine (NE) release from sympathetic nerves is critical for the induction of white fat browning through the β3-adrenergic receptor (β3-AR) since β3-AR loss in mice almost abolished the cold-induced browning in WAT (Galitzky et al., 1995; Jimenez et al., 2003; Collins, 2011). In detail, NE binds to β3-AR and triggers a signal transduction cascade involving cyclic AMP (cAMP)-PKA-CREB signaling, which eventually activates the transcriptions of mitochondrial, lipolytic, lipid oxidative, and thermogenic gene programs (Harms and Seale, 2013; Bargut et al., 2017). CL316,243 (CL), one kind of β3-adrenergic activator, is widely used in cellular models and rodents to mimic cold stimulation with potent effects on metabolic rate and thermogenic effects at least partially via activating browning of white fat since the thermogenic effects of CL still exist in mice lacking brown adipose tissues (Lowell et al., 1993).

Due to the importance of β3-AR signaling in the browning of white fat, the β3-AR agonists are considered a potential therapeutic strategy to combat obesity and metabolic diseases. However, compared to cold, distinct β3-AR agonists featured different efficacies in thermogenic activation and side effects, which complicated its wide and safe use. For example, the applications of various β3-AR agonists have been hindered in clinical use due to their potential adverse effects on the cardiovascular system, which call for continuous efforts for developing novel and safer β3-AR agonists (Arch, 2011). Besides, previous studies have shown that β3-AR stimulation with sympathomimetic ephedrine had no thermogenic effect on human BAT, while mirabegron, a β3-AR agonist used to treat an overactive bladder, was effective in activating BAT as compared to placebo but may have adverse impacts on glucose homeostasis (Cypess et al., 2012, 2015). Moreover, it has been reported that cold exposure and β3-AR agonists may activate distinct cellular populations that express different β-adrenergic receptors (Jiang et al., 2017). Overall, this evidence suggested the obvious heterogeneity of cold exposure and β3-AR agonists on the process of white fat browning, while the detailed differences between two stimulants are not well clarified. A detailed comparative study would offer previously unappreciated mechanisms and strategies for better β3-AR agonists development.

In the present study, via analyzing the transcriptomics of chronic cold exposure or CL administration, we revealed the commonalities and heterogeneity of these two powerful instigators of white fat browning, which may provide novel insights into the theoretical basis in order to optimize β3-AR agonists for the treatment of obesity and metabolic diseases.

## Materials and Methods

### Animals

All of the procedures involving mice were performed according to guidelines of East China Normal University. Male C57BL/6J mice were purchased from Shanghai Model Organisms Center and housed under standard experimental environments controlled at room temperature (22°C) with a 12 h light/dark cycle and free access to food and water. For the establishment of the animal model, mice were housed at either 22°C or 4°C for 7 days in a temperature-controlled incubator (LP-LED, NK system, Japan), or injected daily with 1 mg/kg of PBS or CL316,243 (Sigma-Aldrich) for 7 days. Mice were subsequently sacrificed and inguinal fat was dissected and frozen immediately in liquid nitrogen and stored at −80°C for further analysis. To monitor the effect of the cold or CL treatment, body weight and food intake were measured after 7 days. Serum triglyceride (TG) and total cholesterol (TC) were determined according to the manufacturer’s instructions (Sigma).

### Hematoxylin & Eosin (H&E) and Immunohistochemical (IHC) Staining

Inguinal fat (iWAT) from room temperature (RT) and cold-exposed mice or PBS and CL treated mice were fixed in 10% formalin (Sigma Aldrich). The tissues were embedded into paraffin, blocked, and cut at 5 μm for H&E staining. For UCP1 IHC staining, 5-μm-thick iWAT sections were incubated with 3% H_2_O_2_ to inactivate the endogenous peroxidase and blocked with 5% goat serum for 2 h. Afterward, the slides were incubated with UCP1 antibody (1:200, Abcam) overnight at 4°C and followed with goat-anti rabbit IgG HRP for 1 h. The chromogen DAB was used to detect the immunoreactivity peroxidase. These images were acquired using a microscope (Nikon) and adipocyte sizes were quantified. Briefly, ImageJ software was used to calculate the total area and the number of adipocytes in the whole field of vision, then the sizes were calculated by dividing the total area by the number of adipocytes. Five random fields per section per mouse were analyzed.

### Total RNA Extraction, RNA-Sequencing, and Quantitative Real-Time PCR

Total RNA was extracted from the iWAT of mice with TRIzol (Takara). The RNA library was generated and RNA sequencing (RNA-seq) was performed on an Illumina Hiseq instrument (Cloud-Seq Biotechnology, Shanghai). Reverse transcription and quantitative real-time PCR (qPCR) were performed to confirm the RNA sequencing results. A total of 1 μg of RNA was reversely transcribed using SuperScript^TM^ III Reverse Transcriptase (Takara) for cDNA synthesis, and Universal SYBR Green Master Mix (Yeasen, Shanghai) was used to perform qPCR on the Light cycler 480II machine (Roche, United States). After normalization to 36b4, the relative expression level of RNA was calculated by using the 2^–ΔΔCt^ method.

The sequences of primers are listed in Supplementary Table 2.

### RNA-Seq Analysis of In-House and NCBI Gene Expression Omnibus (GEO) Datasets

A unified method was adopted for all datasets. EdgeR was used to find differentially expressed genes. All sequencing reads were mapped to the mouse reference genome (UCSC mm10) using hisat2 (Kim et al., 2015), and StringTie was used to calculate the read counts (Pertea et al., 2015). EdgeR was used to identify the differentially expressed genes (Robinson et al., 2010). A gene was considered as differentially expressed with the following criteria: *adjusted p-*value < 0.05 and log2| fold change| ≥ 1. Raw datasets have been uploaded to the Gene Expression Omnibus (GSE164219).

To avoid bias or randomization of datasets, we cross-analyzed RNA-seq data from our in-house data GSE164219 and GEO datasets GSE86338 both containing iWAT under room temperature or cold exposure for 7 days, as well as GSE86338 and GSE129083 datasets both for PBS or CL treatment chronologically (Bai et al., 2017; Wang et al., 2019).

### Pathway Enrichment Analysis (KEGG) and Gene Ontology (GO) Analysis

The gene set enrichment analysis (GSEA) was performed to identify the significant pathways and functions using clusterprofiler (Yu et al., 2012). Kyoto Encyclopedia of Genes and Genomes (KEGG) pathway analysis identifies significantly enriched metabolic pathways or signal transduction pathways enriched in differentially expressed genes (DEGs) compared to those with reference gene background using the hypergeometric test. The functional enrichment analysis was carried out to map the DEGs to genes in the GO terms list. The GO terms and KEGG pathways were considered as significantly enriched if *p-*value < 0.05.

### Statistical Analyses

The data analysis was performed with GraphPad Prism 7. The normalcy of data was examined by the Shapiro–Wilk normality test. Statistical comparisons between two groups were made by a two-tailed unpaired student *t*-test. Data are presented as mean ± SEM. Differences between groups were considered statistically significant when *p*-value < 0.05.

## Results

### Chronic Cold Exposure or CL316,243 Administration Induce Browning of White Fat and Thermogenic Marker UCP1 Expression to a Similar Extent in Mice

**FIGURE 1:**
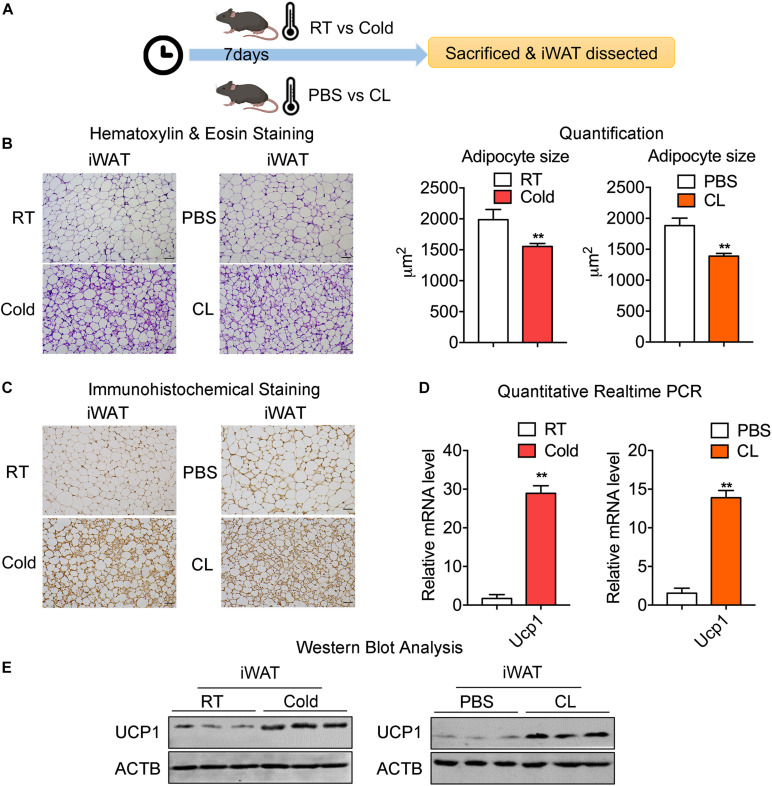
Chronic old exposure and CL316,243 administration induce browning of white fat and thermogenic marker UCP1 levels in mice. **(A)** Schematic illustration of the browning of white fat induced by 7-day cold exposure or β-adrenergic agonist CL316,243 (CL) administration. **(B)** Representative H&E staining of adipocyte size quantification from iWAT of mice treated with RT or cold and PBS or CL. Scale bars: 50 μm. **(C–E)** Representative immunohistochemical images of UCP1 staining **(C)**, mRNA **(D)**, and protein levels of UCP1 **(E)** in iWAT from mice treated with RT or cold and PBS or CL. Scale bars: 50 μm. *N* = 6 per group. Data are presented as mean ± SEM and **P < 0.01 compared to control group.

To confirm the effects of cold exposure or β3-AR agonist stimulation on the browning of white fat, two groups of mice at 8 weeks old were kept under either room temperature (RT, 22°C) or 4°C for 7 days, while the other two groups of mice of a similar age were intraperitoneally (IP) administrated with PBS or CL316,243 (CL) at RT for 7 days (Figure 1A). To explore the effect of the cold or CL treatment on whole-body energy metabolism, we monitored the body weights and food intake changes during cold or CL treatment, as well as triglyceride and cholesterol level after cold or CL treatment. These data showed that both cold and CL treatment reduced body weight and serum lipid parameters (Supplementary Figures 1A–D). Interestingly, cold exposure also exhibited strong effects on increasing food intake compared to CL treatment, which was consistent with previous reports (Yoshida et al., 1996; Jia et al., 2016; Xu et al., 2019). At the end of interventions, inguinal white adipose tissues (iWAT) were dissected from these mice for histological and gene expression analysis (Figure 1A). The hematoxylin & eosin (H&E) staining of iWAT showed reduced adipocyte sizes upon either cold or CL treatment, compared to their respective controls (Figure 1B). In addition, cold and CL both led to a similar strong induction of UCP1, the marker for thermogenesis, at mRNA and protein levels compared to their controls, as shown by qPCR, UCP1 immunohistochemical staining, and western blot analysis (Figures 1C–E). Thus, these results indicated that, physiologically, both cold and CL treatment strongly induced the browning of white fat to a similar extent in mice.

**FIGURE 2:**
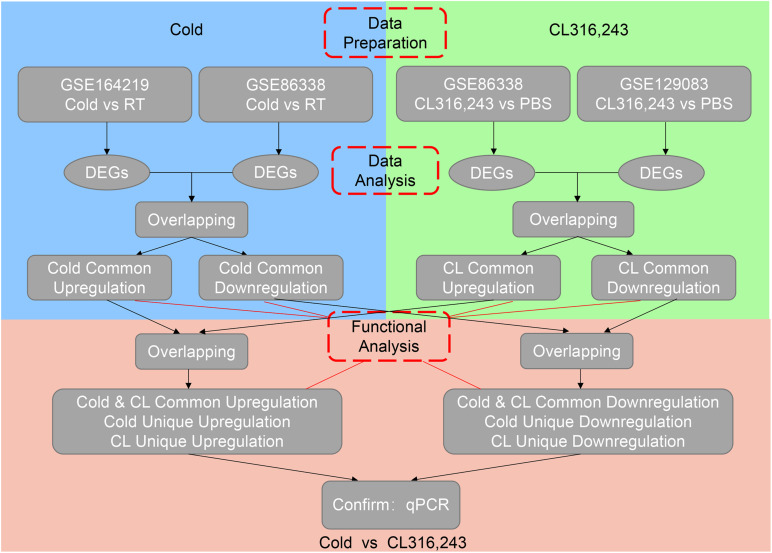
Flow chart of RNA-sequencing data processing.

**FIGURE 3:**
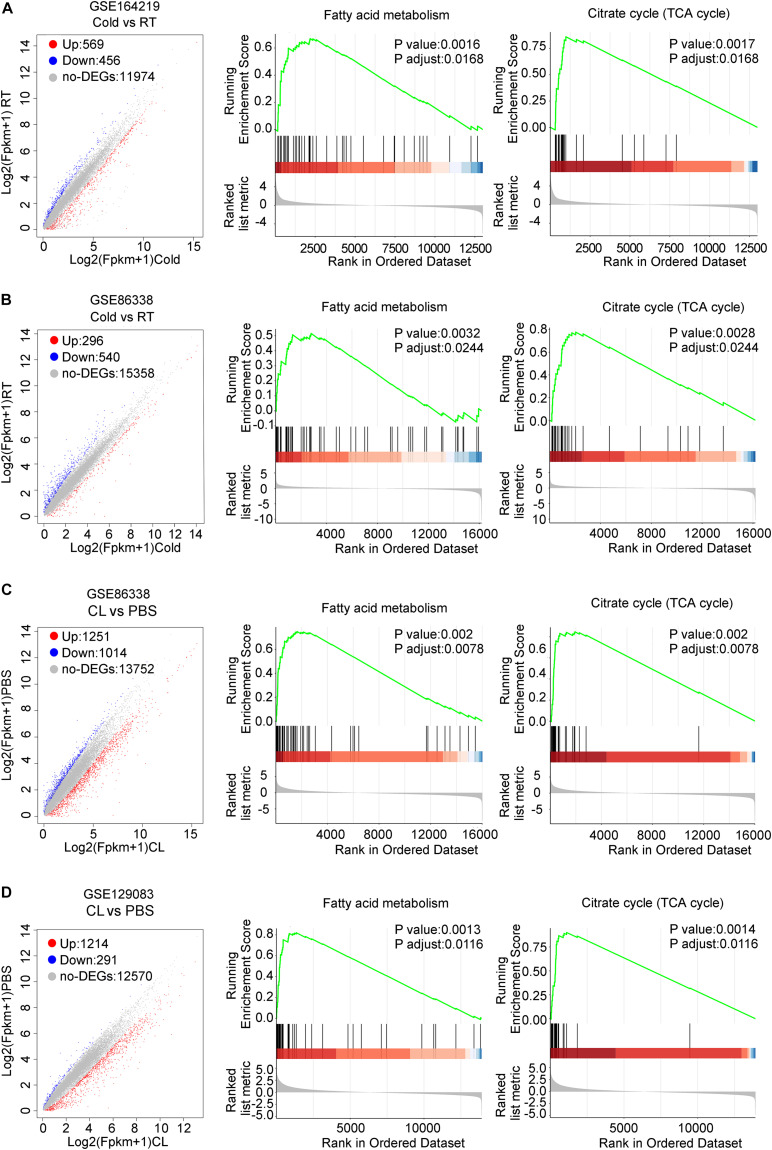
DEGs and GSEA of RNA-seq datasets for iWAT under cold exposure or CL administration in mice. **(A–D)** Volcano plots for DEGs and GSEA of different datasets. **(A)** GSE164219 and **(B)** GSE86338: Cold vs. RT; **(C)** GSE86338 and **(D)** GSE129083: CL316,243 vs. PBS. Red dots for upregulated genes, blue dots for downregulated genes, and gray dots for non-DEGs.

We then set out to analyze the common and differential changes in gene transcriptome during the browning process induced by cold or CL treatment in mice iWAT. Of note, to avoid biased results from a single dataset, we cross-analyzed RNA-seq data using our in-house data GSE164219 and dataset GSE86338 from the GEO database which both analyzed the changes in mRNA landscapes in iWAT under cold exposure or room temperature for 7 days, while the other cross-analysis was performed between datasets GSE86338 and GSE129083 which both evaluated iWAT mRNA changes upon chronic CL or PBS administration (Figure 2). Consistent with a critical role for energy expenditure and thermogenesis in these two browning models, GSEA revealed that cold exposure and CL treatment commonly induced DEGs strongly correlated with fatty acid metabolism and the tricarboxylic acid (TCA) cycle in mitochondria in all datasets (Figures 3A–D).

Overall, these data indicated that cold and CL induced white fat browning featured common characterizations in both fat physiology and critical gene pathways, though the reported differences in functionality and side effects of cold or CL treatment may lie in their individual distinct gene expressions.

### Cold Exposure Enhances Metabolism While Inhibits Inflammatory and Fibrotic Pathways in iWAT

**FIGURE 4:**
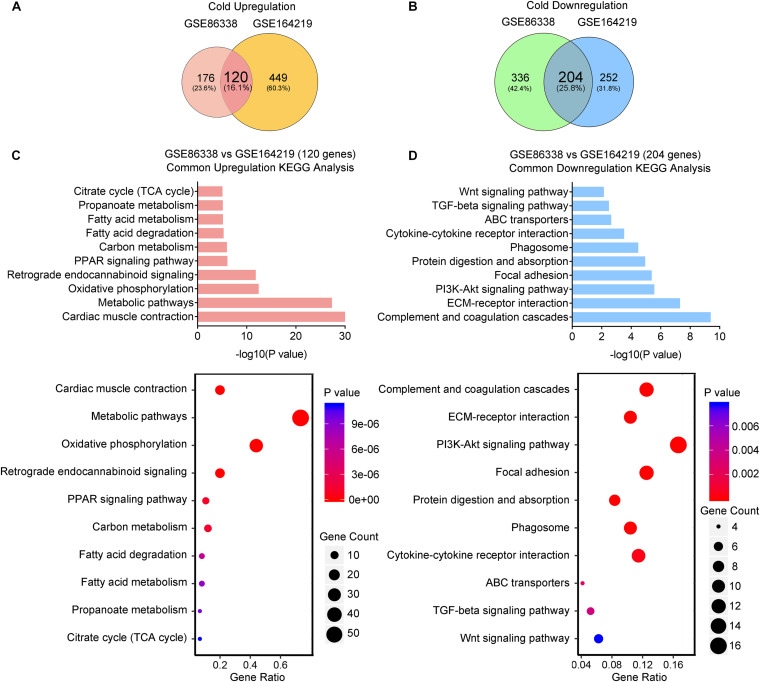
Cold exposure enhances metabolism and inhibits inflammatory and fibrotic pathways in iWAT. **(A,B)** Venn diagram of overlapped upregulated **(A)** and downregulated **(B)** genes from iWAT of mice exposed to cold shown in the GSE86338 and GSE164219 datasets. **(C,D)** KEGG analysis for the DEGs of common upregulated 120 genes **(C)** shown in **(A)** and downregulated 204 genes **(D)** shown in **(B)**.

We thus set out to investigate if cold or CL treatment induce different patterns of gene expressions and pathway enrichments in iWAT. We overlapped two datasets (GSE164219 and GSE86338) to highlight the commonly regulated gene sets upon cold exposure and performed bioinformatic analysis (Overlap-Cold). The results revealed 120 upregulated genes and 204 downregulated genes commonly regulated by cold in both datasets (Figures 4A,B). KEGG analysis revealed that cold stimulated classic metabolic pathways including the adipocyte differentiation-related PPAR signaling pathway, energy homeostasis related carbon metabolism, and TCA cycle (Figure 4C). This was in accordance with GO analysis showing that the lipid metabolic process and mitochondrial regulation were upregulated (Supplementary Figure 2A). Notably, in our analysis, cold exposure induced gene programs enriched in cardiac muscle contraction, suggesting that chronic cold may also pose potential threats to the cardiovascular system. Meanwhile, KEGG analysis also revealed that cold exposure inhibited complement and coagulation cascades, ECM-receptor interaction, and the TGF-β signaling pathway (Figure 4D), and GO analysis highlighted that the regulated genes were involved in extracellular matrix organization (Supplementary Figure 2B), which potentially contributed to adipose tissue fibrosis (Sun et al., 2013).

Taken together, these transcriptional data suggested that cold exposure promoted classic energy metabolism while it suppressed the inflammatory and fibrotic pathways in iWAT.

### CL Stimulates a Broader Spectrum of Metabolic Gene Pathways and Decreases Immune Responses

**FIGURE 5:**
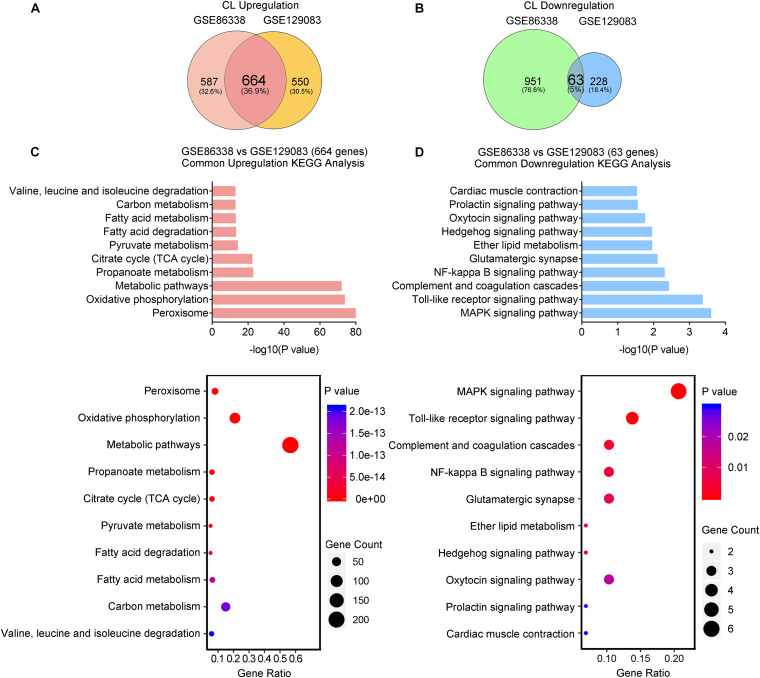
CL stimulates a broader spectrum of metabolic gene pathways and decreases immune responses. **(A,B)** Venn diagram of overlapped upregulated **(A)** and downregulated **(B)** genes from iWAT of mice administrated with CL shown in GSE86338 and GSE129083 datasets. **(C,D)** KEGG analysis for the DEGs of common upregulated 664 genes **(C)** shown in **(A)** and downregulated 63 genes **(D)** shown in **(B)**.

We also overlapped genes from two datasets (GSE86338 and GSE129083) that study CL treatment, which rendered 664 commonly upregulated and 63 commonly downregulated genes (Overlap-CL, Figures 5A,B). Interestingly, the counts of commonly upregulated genes treated with CL were far more than those found in cold, suggesting that CL may cause changes to a broader spectrum of gene programs in iWAT (Figures 5A,C). Indeed, in addition to fatty acid metabolism and TCA cycle, KEGG analysis uncovered that CL positively regulated peroxisome and pyruvate metabolism, suggesting other cellular organelles and glucose metabolism were mobilized by CL treatment (Figure 5C). Consistently, GO analysis also showed that the upregulated DEGs were associated with lipid and carbohydrate metabolism (Supplementary Figure 3A). In comparison, only 5% of commonly downregulated genes were affected, including immune response pathways such as the toll-like receptor signaling pathway and NF-kappa B signaling pathway (Figure 5D), which were consistent with GO analysis results showing a suppressed acute inflammatory response and IL-8 production (Supplementary Figure 3B).

Therefore, our transcriptional analysis revealed that aside from the classic energy metabolism observed in cold stimulation, CL treatment also induced peroxisomal and pyruvate metabolism, while subduing classic immune responses.

### Comparative Analysis Revealed That Cold and CL Exposure Commonly Increase Oxidative Phosphorylation and Metabolic Pathways and Decrease Complement and Coagulation Cascades

**FIGURE 6:**
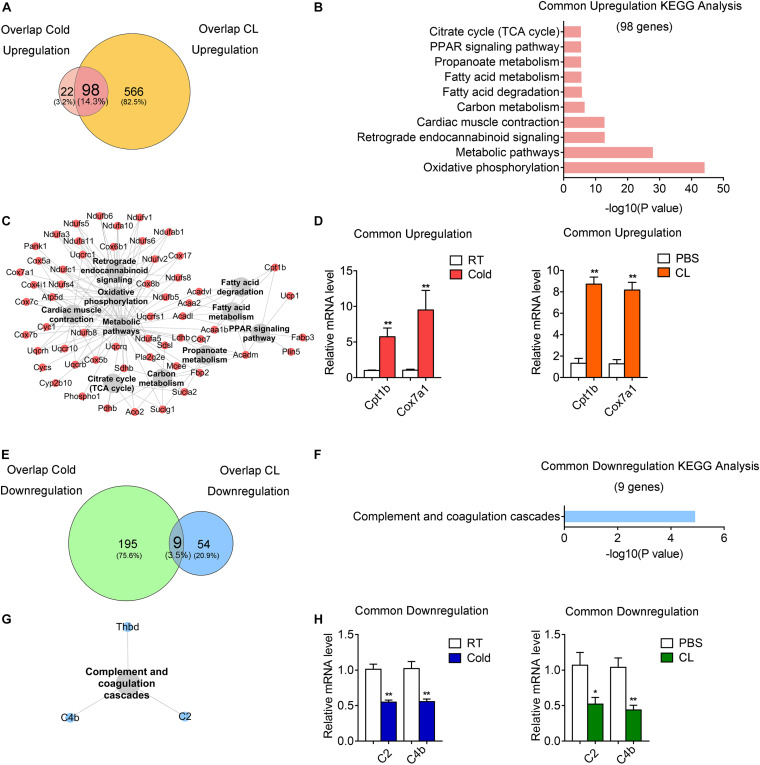
Comparative analysis of commonly regulated genes by cold and CL exposure. Analysis of overlapped DEGs between cold (commonly regulated genes from GSE86338 and GSE164219 datasets) and CL (commonly regulated genes from GSE86338 and GSE129083 datasets). **(A,E)** Venn diagrams for commonly upregulated **(A)** and downregulated **(E)** DEGs between cold and CL treatment. **(B,C,F,G)** KEGG analysis and pathway-gene network for the upregulated 98 genes **(B,C)** shown in **(A)** and downregulated 9 genes **(F,G)** shown in **(E)**. **(D,H)** mRNA levels of top ranked upregulated **(D)** genes from **(A)** and downregulated **(H)** genes from **(E)**. *N* = 6 per group. Data are presented as mean ± SEM and *P < 0.05, **P < 0.01 compared to control group.

Subsequently, we investigated the commonality between cold and CL treatment by further cross-analyzing the overlap-cold and overlap-CL datasets. Intriguingly, the majority of upregulated genes under the cold condition were covered by DEGs set under CL treatment (Figure 6A). The 98 genes commonly upregulated by cold and CL were majorly involved in oxidative phosphorylation and the metabolic pathways required for thermogenesis as revealed by KEGG analysis (Figures 6B,C). Furthermore, among the top enriched genes were classic thermogenic and mitochondrial genes including Ucp1, Cpt1b, and Cox7a1, which were confirmed by qPCR analysis (Supplementary Table 1 and Figures 1D, 6D). On the contrary, only nine genes, mainly related to complement and coagulation cascades, were commonly downregulated by cold and CL, with complement components C2 and C4b confirmed by qPCR (Figures 6E–H). Complement components are a critical part of the innate immune system and contribute to excessive inflammatory responses (Lin et al., 2018; Lung et al., 2019).

These results indicated that both cold and CL treatment promoted thermogenesis and energy metabolism via oxidative phosphorylation, while they suppressed complement and coagulation cascades.

### Analysis of Unique Gene Programs of Cold Exposure and CL Treatment Suggest Distinct Energy Substrates Utilization and Physiological Events

Lastly but most importantly, we explored the heterogeneity in gene expression patterns between cold exposure and CL treatment during iWAT browning. Notably, after overlapping datasets, we found that 3.2% of DEGs (22 genes) were uniquely upregulated by cold, while 82.5% of DEGs (566 genes) were uniquely upregulated by CL (Figure 6A). These results suggested that though cold and CL both strongly induce white fat browning, CL regulates a unique set of gene programs compared to cold, which may explain the different functionality and side effects induced by these two stimulations.

**FIGURE 7:**
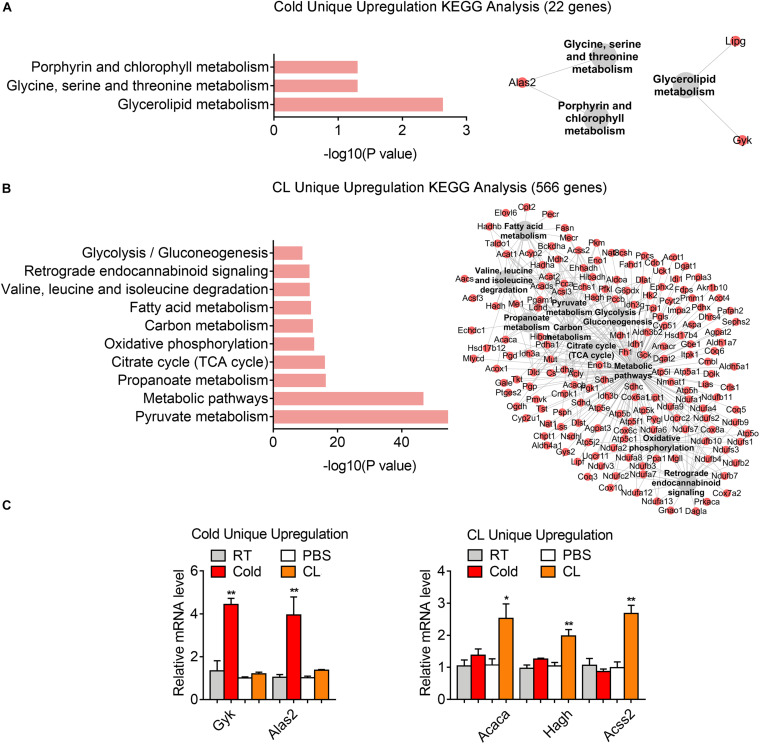
Analysis of uniquely upregulated gene programs of cold exposure and CL administration. **(A,B)** KEGG analysis and pathway-gene network for uniquely upregulated DEGs [**(A)** 22 genes in cold datasets, **(B)** 566 genes in CL datasets]. **(C)** mRNA levels of top ranked genes exclusively upregulated in iWAT of mice under cold exposure or CL administration. *N* = 6 per group. Data are presented as mean ± SEM and *P < 0.05, **P < 0.01 compared to control group.

Kyoto Encyclopedia of Genes and Genomes analysis revealed a significant enrichment of glycerolipid metabolism, as well as glycine, serine, and threonine metabolism as a feature of cold-induced gene patterns (Figure 7A). qPCR analysis confirmed the increase in mRNA levels of aminolaevulinic acid synthase 2 (ALAS2), the rating-limiting enzyme in heme synthesis which can maintain the mitochondrial function in brown adipocytes (Galmozzi et al., 2019), as well as glycerol kinase (Gyk), which catalyzes the phosphorylation of glycerol to glycerol 3-phosphate and is reported to be involved in UCP1 level induction (Iwase et al., 2020), in cold-treated iWAT versus CL treatment (Figure 7C). Meanwhile, we noted that pyruvate metabolism, carbon metabolism, and glycolysis were uniquely enriched in CL treatment, suggesting that in addition to oxidative phosphorylation, fatty acid metabolism, and TCA cycle that were commonly regulated by cold and CL, CL treatment features active glucose mobilization for energy demands (Figure 7B). Among the top regulated genes, qPCR analysis confirmed enhanced expression of acetyl-CoA carboxylase alpha (ACACA), hydroxyacylglutathione hydrolase (HAGH), and acyl-CoA synthetase short chain family member 2 (ACSS2) in the pyruvate catabolism (Figure 7C) in CL-treated iWAT versus cold. These results suggested that cold uniquely regulated glycerolipid metabolism and specific amino acids metabolism, while compared to cold, CL treatment tended to mobilize both carbon and lipid substrates for the browning of white fat.

**FIGURE 8:**
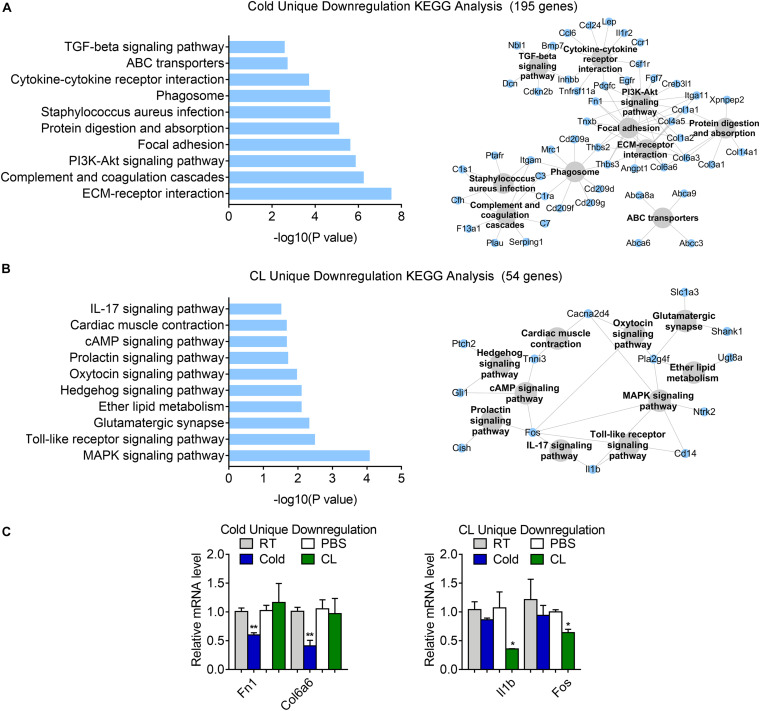
Analysis of uniquely downregulated gene programs of cold exposure and CL administration. **(A,B)** KEGG analysis and pathway-gene network for uniquely downregulated DEGs [**(A)** 195 genes in cold datasets, **(B)** 54 genes in CL datasets]. **(C)** mRNA levels of top ranked genes exclusively downregulated in iWAT of mice under cold exposure or CL administration. *N* = 6 per group. Data are presented as mean ± SEM and *P < 0.05, **P < 0.01 compared to control group.

Besides, 75.6% of DEGs (195 genes) were uniquely downregulated upon cold exposure while 20.9% of DEGs (54 genes) were uniquely suppressed by CL treatment (Figure 6E), suggesting that cold suppressed a large number of distinct genes compared to CL. KEGG analysis showed that cold exposure mainly inhibited ECM-receptor interaction and focal adhesion, while CL inhibited pathways included the MAPK signaling pathway, toll-like receptor signaling pathway, and glutamatergic synapse (Figures 8A,B). Besides, the unique genes suppressed by cold or CL treatment were confirmed by qPCR, including fibrotic and collagen related genes Fn1, Col6a6 regulated by cold, as well as Fos and proinflammatory cytokine IL-1b regulated by CL (Figure 8C). These results indicated that cold treatment tended to downregulate ECM-related pathways, while CL treatment decreased specific intracellular and receptor signaling pathways.

Altogether, our analyses revealed different energy substrates utilization and specific physiological events induced by cold or CL stimulated in beige adipocytes.

## Discussion

Chronic cold exposure and β3-AR treatment induce transcriptional signaling pathways in beige adipocytes for thermogenesis. In the present study, we examined the transcriptomic alterations that occur in the iWAT of mice during cold exposure or CL treatment by analyzing overlapped RNA-seq datasets and revealed that these two triggers of browning commonly drove lipid metabolism and TCA cycle in mitochondria for thermogenesis. Interestingly, we found differences between datasets generated from mice under similar treatment, which may be caused by multiple factors behind this phenomenon. First, the mice strains used by different labs may have differences due to sub-strains formed by long-term inbreeding in different facilities. Second, it has been more and more recognized that housing environments may have a major influence on experimental outcomes, including different diet formula and bedding materials that the mice are kept on, distinct circadian rhythm due to different lighting schedules in the facility, or different times when the experiments were performed, etc. Last but not least, gut microbiota in animals from different facilities are different, which may contribute to the different metabolic performances and responsive gene programs under cold or CL treatment. To circumvent this factor, we overlapped different datasets to identify the stable alternations of gene programs between datasets under cold or CL treatment. Of note, CL treatment uniquely activated carbohydrates mobilization for energy demands, and cold uniquely promoted glycerolipid and specific amino acids metabolism. Meanwhile, we found that cold and CL treatment each suppressed different inflammatory events, while cold additionally downregulated fibrotic programs. These results may provide novel insights for understanding the molecular mechanisms behind the functional differences and distinct side effects caused by cold or β3-AR agonist stimulation in the browning process.

We found that both cold and CL stimulation on beige fat significantly enhanced pathways related to oxidative phosphorylation for energy metabolism. White adipose tissue mainly stores energy in the form of triglycerides (TG). In the face of energy demand, for example, during cold or CL stimulation, TG breaks into glycerol and fatty acid (Beale et al., 2002) to provide energy substrates. Via transcriptome data and qPCR confirmation, we confirmed that Gyk, an enzyme critical for glycerol/fatty acid metabolism and UCP1 induction partially through the β3AR-cAMP-CREB pathway (Iwase et al., 2020), was upregulated upon cold exposure, indicating a critical role of lipid metabolism in cold-stimulated browning. Besides, we found that compared to CL treatment, cold exposure specifically promoted glycerolipid metabolism that is an integral part of the glycerolipid/FFA cycle essential for maintaining body temperature by releasing heat at the expense of ATP (Prentki and Madiraju, 2008). Since we found changes in glycerolipid metabolism uniquely in cold treatment but not CL treatment, it is possible that cold exposure also promoted thermogenesis by enhancing glycerolipid metabolism in a β3-AR-independent way. In addition, an interesting observation from our transcriptome data was that the upregulated DEGs in CL-induced browning included the majority of upregulated DEGs of cold-induced browning, while CL also featured unique DEGs, i.e., pyruvate metabolism among the top ranking pathway categories by KEGG analysis. This indicates that cold and CL activated an array of similar gene pathways, i.e., lipid metabolism for substrate consumption, while CL additionally promoted multiple substrate utilization, for example, glucose metabolism for enhanced oxidation phosphorylation, suggesting that CL was more prone to mobilize both carbohydrate and lipids for adequate heat production.

It is well known that proinflammatory macrophages infiltrate adipose tissues in obese mice, which leads to chronic low-grade systemic inflammation and obesity-related metabolic syndrome (Weisberg et al., 2003; Lumeng and Saltiel, 2011; Nguyen et al., 2011). This local pro-inflammatory environment in fat tissues directly impairs thermogenic activity, which also impacts brown-versus-white plasticity in subcutaneous adipose tissue (Villarroya et al., 2018). Meanwhile, thermogenic stimuli such as cold exposure and β3-AR agonists treatment, have been reported to protect against metabolic derangements in obesity partially via triggering anti-inflammatory responses during tissue remodeling and beige adipogenesis (Lee et al., 2013; Hui et al., 2015; Burl et al., 2018). In our study, among the uniquely downregulated gene pathways in cold, we found that fibrotic genes, such as the fibronectin Fn1 and Col family involved in ECM-receptor interaction, were highlighted in the top ranking gene list (Supplementary Table 1), indicating that tissue remodeling is a critical event during cold-induced white fat browning. It is known that adipocytes undergo dramatic expansion during strong obesogenic insults, whereas collagen families are key factors in maintaining ECM integrity and promoting ECM remodeling (Khan et al., 2009; Mariman and Wang, 2010; Villarroya et al., 2018). Col4a5, Col6a6, and Fn1 genes in charge of producing collagen of ECM components and markers of iWAT fibrosis, were discovered to be uniquely decreased in cold (Zhang et al., 2020). Therefore, our results indicated that cold exposure could relieve adipose tissue fibrosis, which is one of the major characteristics for adipose tissue aging, thus offering a potential therapeutic method for treating aging-associated metabolic decline. Besides, the adaptive immune system is activated along with the development of adipose tissue inflammation during adipocytes hypertrophy (Kintscher et al., 2008). In this study, we found that interleukin family member IL-1b and Fos, a nucleoprotein transcription factor reported to orchestrate the functions of interleukin-family such as IL-17 and IL-1b for inflammatory responses (Frohnert et al., 2014; Camarena et al., 2017), were downregulated upon CL administration. It is thus interesting that although both cold and CL inhibit inflammation in iWAT, they impact different immune signaling pathways and inflammatory genes, which may provide a theoretical basis for studies on the interactions between obesity and inflammation.

Recently, single cell RNA sequencing (scRNA-seq) and single nucleus RNA-seq (snRNA-seq) have emerged as powerful tools to dissect tissue heterogeneity, which have been applied to the studies of development and function of adipose tissue (Burl et al., 2018; Rajbhandari et al., 2019; Henriques et al., 2020; Sun et al., 2020). These studies characterize various cell populations in SVF or mature adipocytes of iWAT as well as changes in immune cells including the upregulated expression of IL-10 and increased ratio of M2/M1 macrophages during iWAT browning process via scRNA-seq and snRNA-seq, which was similar to our result that both cold exposure and CL treatment affected the immune response pathways (Rajbhandari et al., 2019; Henriques et al., 2020). However, few single-nuclei transcriptome analyses have focused on the different impacts between cold and CL treatment on iWAT, which warrants further study. Besides, epigenetic modification has been demonstrated to play a critical role in regulating the development and function of adipose tissue. For example, upon activating β3-AR signaling, JMJD1A-mediated H3K9me2 demethylation promotes beige fat adipogenesis and KMT5c-mediated H4K20 methylation activates thermogenic gene program in iWAT (Abe et al., 2018; Zhao et al., 2020). In addition, studying different protein characteristics would offer additional dimensions in interpreting the differential responses caused by cold or CL stimulation. In this study, we explored the commonalities and heterogeneity of cold exposure and CL administration on iWAT transcriptome during the induction of beige fat browning. Future studies on how different stimuli convey different changes in epigenetic regulation, cell subsets, or proteomics within iWAT remain to be further explored.

Besides adipose tissues, liver, and skeletal muscle may also play important roles in regulating systemic energy homeostasis in response to cold or CL treatments (Baskin et al., 2015). In acute cold, muscles provide heat for body temperature defenses through shivering and non-shivering thermogenesis. For example, Sarcolipin (Sln) has been shown to be an important mediator in muscle-based non-shivering thermogenesis. Under acute cold exposure, Sln knock-out mice failed to maintain their core body temperatures (Bal et al., 2012). Meanwhile, CL treatment has been shown to promote the expression of uncoupling proteins such as UCP2 and UCP3 in skeletal muscle (Nagase et al., 1996; Nakamura et al., 2001). Besides, it has been reported that cold stimulation promoted FFA release from white adipocytes, which caused a metabolic switch in the liver by activating the nuclear receptor HNF4α and producing acylcarnitines (Simcox et al., 2017). Here, we aimed to explore the commonalities and heterogeneity of cold exposure and CL administration on iWAT transcriptome during the induction of beige fat browning, though contributions from other metabolic organs may also play a role in the overall outcomes.

## Conclusion

In summary, our results specify both common and unique features of the molecular signatures in white fat brown activated by cold exposure or CL treatment. They commonly activate mitochondrial gene programs. Moreover, both stimulations inhibit inflammation and cause physiological changes in cellular components, though with different preferences. In terms of substrate utilization, both stimulants mobilize lipid metabolism for heat production, while CL is additionally prone to utilize carbohydrates for energy demands. Overall, our data offer novel insights toward the complex molecular events induced by various thermogenic stimulants, and provides further understanding of the thermogenic mechanism and physiological application of β3-AR agonists.

## Data Availability Statement

The datasets presented in this study can be found in online repositories. The names of the repository/repositories and accession number(s) can be found in the article/Supplementary Material.

## Ethics Statement

The animal study was reviewed and approved by the East China Normal University.

## Author Contributions

XM, DW, and LX conceived and designed the experiments, and wrote and revised the manuscript. YL, XP, and YZ performed the bioinformatic analysis and established the animal models. GL and TZ assisted in the experiments. GC assisted in data analysis. All authors have read and agreed to the published version of the manuscript.

## Conflict of Interest

The authors declare that the research was conducted in the absence of any commercial or financial relationships that could be construed as a potential conflict of interest.

## Supplementary Material

Supplementary Figure 1 Effect of chronic cold exposure and CL316,243 administration on systemic energy homeostasis. **(A–D)** Body weight gain, food intake, serum triglyceride (TG), and total cholesterol (TC) levels of mice treated with RT or cold **(A,B)** and PBS or CL **(C,D)**. *N* = 6 per group. Data are presented as mean ± SEM and ^∗∗^*P* < 0.01 compared to control group.

Supplementary Figure 2 Gene ontology (GO) biological process analysis of commonly regulated genes in iWAT of mice upon cold exposure from two RNA-seq datasets. **(A,B)** GO enrichment analysis for overlapped upregulated **(A)** and downregulated **(B)** DEGs from iWAT of mice under cold exposure as shown in GSE86338 and GSE164219 datasets.

Supplementary Figure 3 Gene ontology (GO) biological process analysis of commonly regulated genes in iWAT of mice upon CL administration from two RNA-seq datasets. **(A,B)** GO analysis for overlapped upregulated **(A)** and downregulated **(B)** DEGs from iWAT of mice under CL administration as shown in GSE86338 and GSE129083 datasets.
