# Supplementary material for: Comparative Transcriptome Profiling of Cold Exposure and β3-AR Agonist CL316,243-Induced Browning of White Fat
Source: Front Physiol. 2021 May 4;12:667698. doi: 10.3389/fphys.2021.667698 (PMC8129586; doi:10.3389/fphys.2021.667698)
Supplement: Supplementary Figure 1 — Effect of chronic cold exposure and CL316,243 administration on systemic energy homeostasis. (A–D) Body weight gain, food intake, serum triglyceride (TG), and total cholesterol (TC) levels of mice treated with RT or cold (A,B) and PBS or CL (C,D). N = 6 per group. Data are presented as mean ± SEM and ∗∗P < 0.01 compared to control group. [file Data_Sheet_1.PDF]

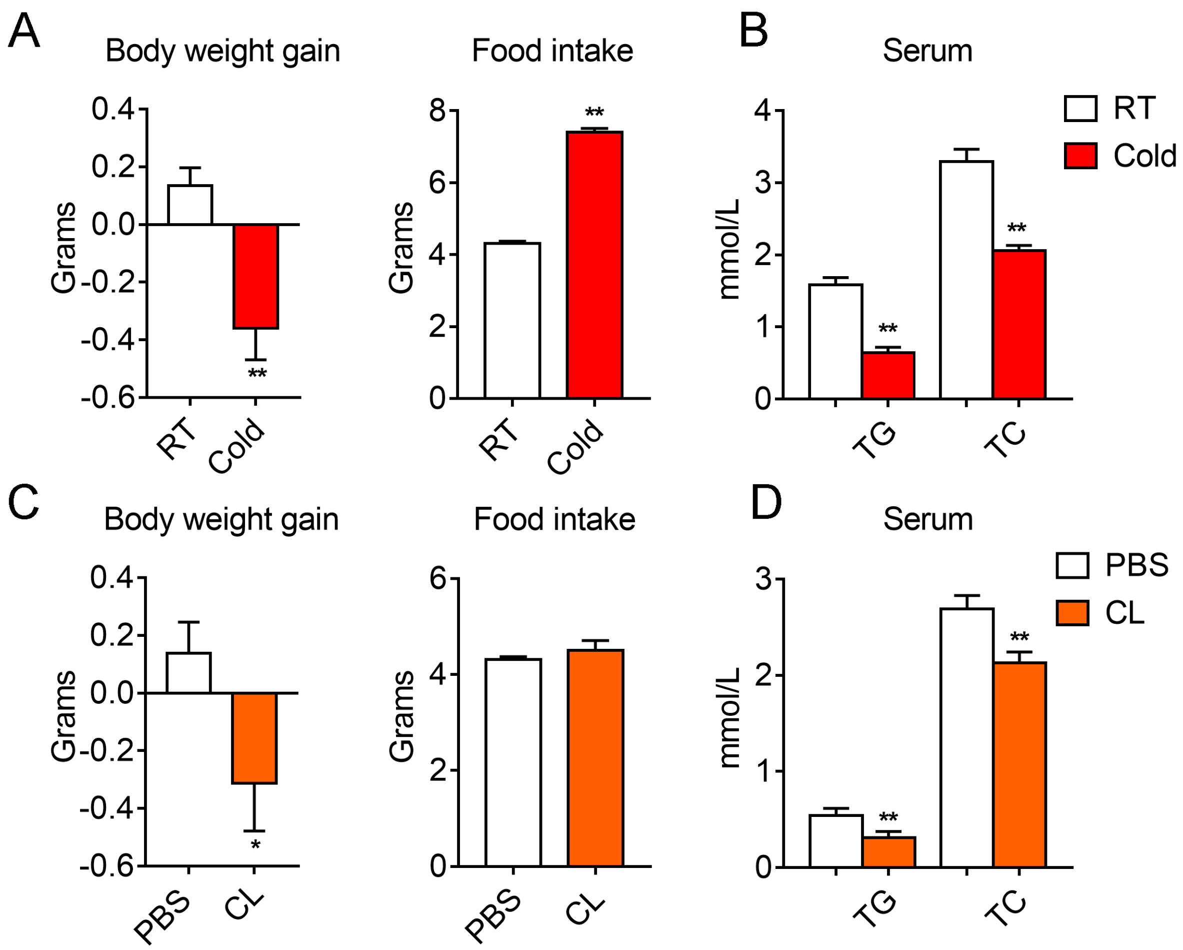

A
Body weight gain
Grams
0.4
0.2
0.0
-0.2
-0.4
-0.6
**
RT
Cold
Food intake
Grams
8
6
4
2
0
**
RT
Cold
B
Serum
mmol/L
4
3
2
1
0
**
**
TG
TC
RT
Cold
C
Body weight gain
Grams
0.4
0.2
0.0
-0.2
-0.4
-0.6
*
PBS
CL
Food intake
Grams
6
4
2
0
PBS
CL
D
Serum
mmol/L
3
2
1
0
**
**
TG
TC
PBS
CL

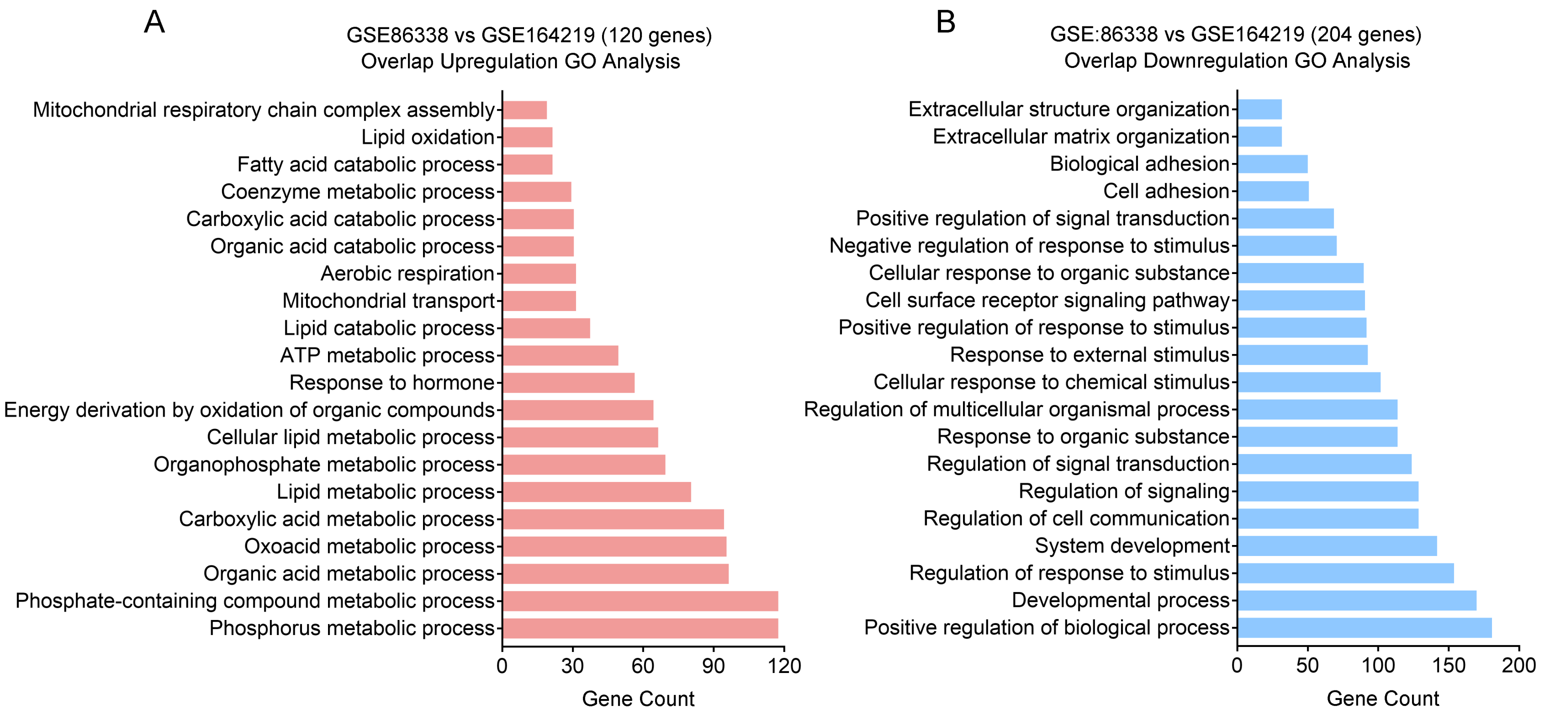
A
GSE86338 vs GSE164219 (120 genes)
Overlap Upregulation GO Analysis
Mitochondrial respiratory chain complex assembly
Lipid oxidation
Fatty acid catabolic process
Coenzyme metabolic process
Carboxylic acid catabolic process
Organic acid catabolic process
Aerobic respiration
Mitochondrial transport
Lipid catabolic process
ATP metabolic process
Response to hormone
Energy derivation by oxidation of organic compounds
Cellular lipid metabolic process
Organophosphate metabolic process
Lipid metabolic process
Carboxylic acid metabolic process
Oxoacid metabolic process
Organic acid metabolic process
Phosphate-containing compound metabolic process
Phosphorus metabolic process
0
30
60
90
120
Gene Count
B
GSE:86338 vs GSE164219 (204 genes)
Overlap Downregulation GO Analysis
Extracellular structure organization
Extracellular matrix organization
Biological adhesion
Cell adhesion
Positive regulation of signal transduction
Negative regulation of response to stimulus
Cellular response to organic substance
Cell surface receptor signaling pathway
Positive regulation of response to stimulus
Response to external stimulus
Cellular response to chemical stimulus
Regulation of multicellular organismal process
Response to organic substance
Regulation of signal transduction
Regulation of signaling
Regulation of cell communication
System development
Regulation of response to stimulus
Developmental process
Positive regulation of biological process
0
50
100
150
200
Gene Count

A

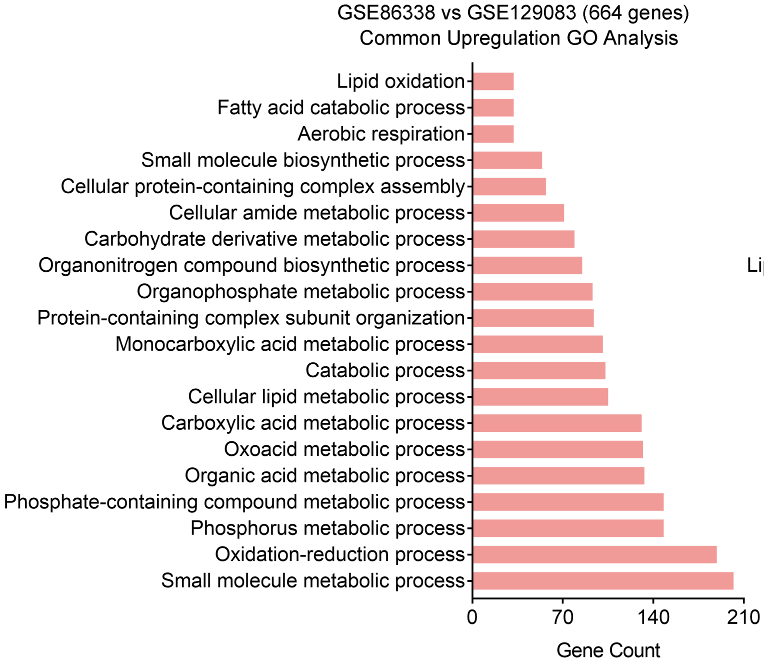

GSE86338 vs GSE129083 (664 genes)
Common Upregulation GO Analysis
Lipid oxidation
Fatty acid catabolic process
Aerobic respiration
Small molecule biosynthetic process
Cellular protein-containing complex assembly
Cellular amide metabolic process
Carbohydrate derivative metabolic process
Organonitrogen compound biosynthetic process
Organophosphate metabolic process
Protein-containing complex subunit organization
Monocarboxylic acid metabolic process
Catabolic process
Cellular lipid metabolic process
Carboxylic acid metabolic process
Oxoacid metabolic process
Organic acid metabolic process
Phosphate-containing compound metabolic process
Phosphorus metabolic process
Oxidation-reduction process
Small molecule metabolic process
0
70
140
210
Gene Count

B

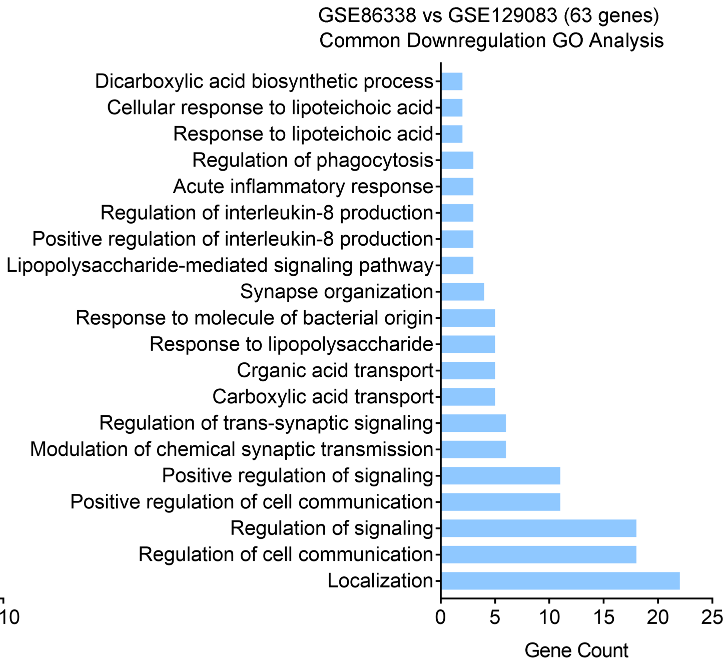

GSE86338 vs GSE129083 (63 genes)
Common Downregulation GO Analysis
Dicarboxylic acid biosynthetic process
Cellular response to lipoteichoic acid
Response to lipoteichoic acid
Regulation of phagocytosis
Acute inflammatory response
Regulation of interleukin-8 production
Positive regulation of interleukin-8 production
Lipopolysaccharide-mediated signaling pathway
Synapse organization
Response to molecule of bacterial origin
Response to lipopolysaccharide
Crganic acid transport
Carboxylic acid transport
Regulation of trans-synaptic signaling
Modulation of chemical synaptic transmission
Positive regulation of signaling
Positive regulation of cell communication
Regulation of signaling
Regulation of cell communication
Localization
0
5
10
15
20
25
Gene Count
